# Bio++: a set of C++ libraries for sequence analysis, phylogenetics, molecular evolution and population genetics

**DOI:** 10.1186/1471-2105-7-188

**Published:** 2006-04-04

**Authors:** Julien Dutheil, Sylvain Gaillard, Eric Bazin, Sylvain Glémin, Vincent Ranwez, Nicolas Galtier, Khalid Belkhir

**Affiliations:** 1 CNRS UMR 5171 – Génome, Populations, Interactions, Adaptation (GPIA), Université Montpellier 2, France; 2 CNRS UMR 5554 – Institut des Sciences de I'Evolution de Montpellier (ISE-M), Université Montpellier 2, France

## Abstract

**Background:**

A large number of bioinformatics applications in the fields of bio-sequence analysis, molecular evolution and population genetics typically share input/ouput methods, data storage requirements and data analysis algorithms. Such common features may be conveniently bundled into re-usable libraries, which enable the rapid development of new methods and robust applications.

**Results:**

We present Bio++, a set of Object Oriented libraries written in C++. Available components include classes for data storage and handling (nucleotide/amino-acid/codon sequences, trees, distance matrices, population genetics datasets), various input/output formats, basic sequence manipulation (concatenation, transcription, translation, etc.), phylogenetic analysis (maximum parsimony, markov models, distance methods, likelihood computation and maximization), population genetics/genomics (diversity statistics, neutrality tests, various multi-locus analyses) and various algorithms for numerical calculus.

**Conclusion:**

Implementation of methods aims at being both efficient and user-friendly. A special concern was given to the library design to enable easy extension and new methods development. We defined a general hierarchy of classes that allow the developer to implement its own algorithms while remaining compatible with the rest of the libraries. Bio++ source code is distributed free of charge under the CeCILL general public licence from its website .

## Background

The design of re-usable software components into libraries has proved to be useful to the rapid development of bioinformatics applications, as witnesses the success of the ever growing open source libraries known as the Bio{*} projects. These libraries cover many fields of bioinformatics, but rarely offer tools for intensive calculus, as required for phylogenetic analyses for instance. Such computer-expensive applications are usually written in C, a compiled low-level language. Programming time-consuming tasks often requires the implementation of specific algorithms optimized for each particular problem. However, despite these specifities, there are some common points (*e.g. *likelihood calculation, minimization), and developers will appreciate to use modules pre-programmed in a flexible manner.

The use of the C++ language combines both the computer efficiency of the C language and the convenience of a Object-Oriented (OO) approach. We present Bio++, a set of C++ libraries dedicated to sequence analysis, molecular evolution and population genetics. Other libraries taking these advantages of C++ have been developed, including the libsequence library [1] for population genetics analysis (single nucleotides polymorphism and coalescent), PDBlib for manipulating tridimensional structures [2] or libcov [3] for protein phylogenetics and molecular evolution. The Bioinformatics Template Library [4] also provides algorithms for manipulating and analysing three dimensional structures, using a generic programming approach.

## Implementation

We did a collaborative effort to design a hierarchy of useful classes for the rapid development of efficient applications in the fields of sequence analysis, phylogenetics, molecular evolution and population genetics. These libraries combine tools for data handling and numerical calculations, providing an easy-to-use, powerful and general development environment. The three main objectives of Bio++ are (i) to speed up the implementation of a new method in order to test it, (ii) to make easier the development of robust applications for distribution and (iii) to provide a high level programming language for biologists.

### Library design

We used object-oriented programming paradigms to develop Bio++. We defined for each class an abstract basal class that contains a number of purely virtual functions (dummy functions with an empty boddy). Such a basal class is called an interface and can be seen has a kind of contract that a class must follow in order to be used by other methods. Such a contract only specifies what an object is at least able to do, and not how it does it. Most classes in Bio++ are derived from interfaces, and some common instanciations are proposed for each interface. The user may, however, write its own implementation of a given method, and still remain compatible with the rest of the library. For instance, one may easily design a new alphabet to handle a new kind of sequences, or write a new substitution model and perform likelihood calculation without writing any likelihood computation function. Conversely, one may implement a new likelihood computation algorithm without re-developping substitution model classes. Several interfaces and abstract classes (partial implementations) linked by inheritance specify different levels of specialization. Depending on their objectives and programming level, users might simply use fully-specified objects, re-implement specific methods, or even design new classes.

**Figure 1:**
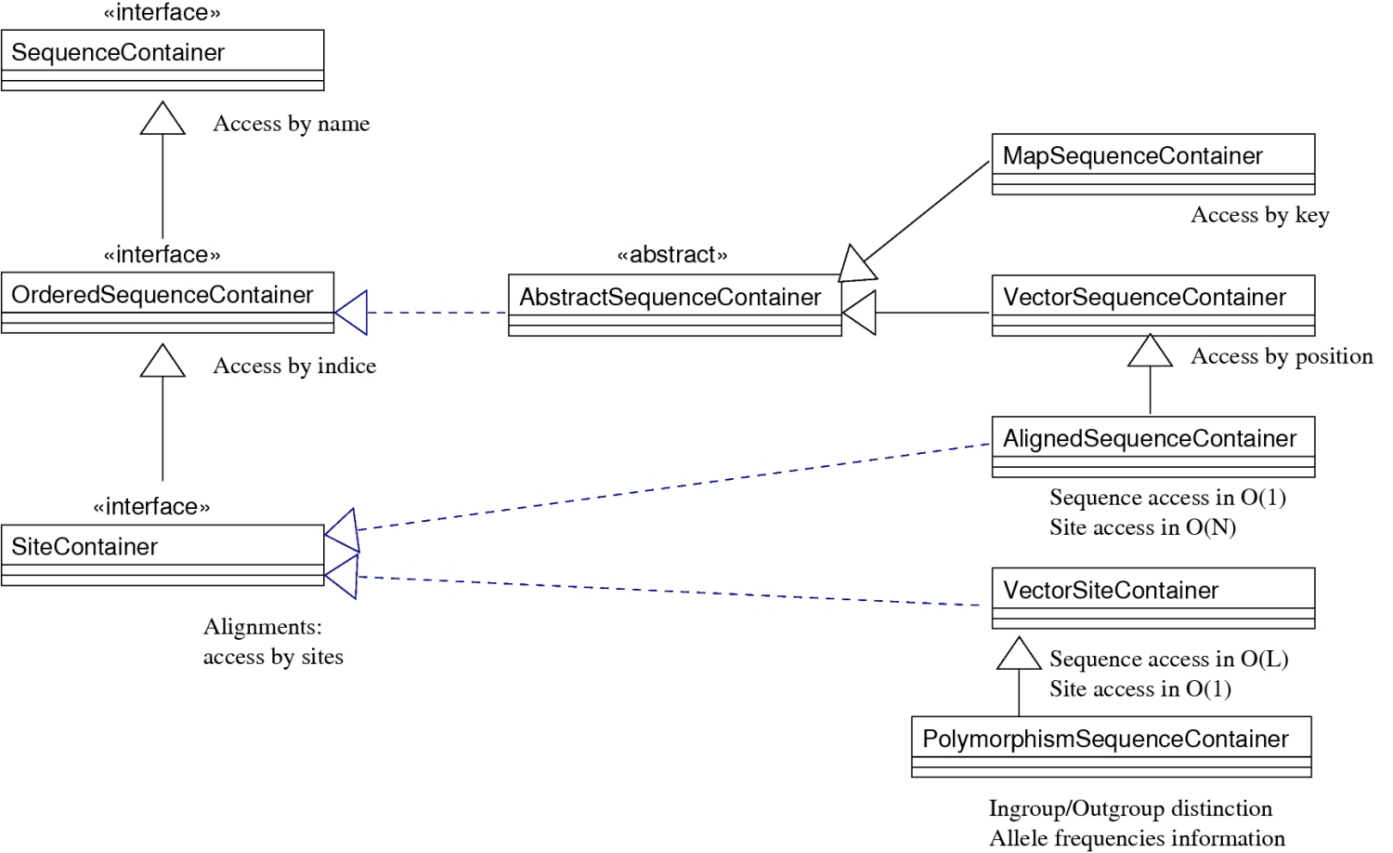
**Class hierarchy for the sequence container family. **"Interfaces" are classes with only pure virtual methods. "Abstract" classes are partial implementations of interfaces. Full arrows indicate inheritence links and dashed arrows represent implementation links.

The sequence container hierarchy is a representative example of the class hierarchies defined in Bio++ (see figure 1). The SequenceContainer basal interface only specifies that sequences can be accessed by their name. The OrderedSequenceContainer has the additional requirement that sequences can be accessed by index, which is generally the case, even if ordering has no biological meaning. The SiteContainer interface requires that sequences have the same length (*i.e. *are aligned) and hence may also be accessed by site (= column in the alignment). Several implementations are proposed: in the VectorSequenceContainer class, data are stored as a vector of sequences. Each sequence can hence be accessed in *O*(1). If there are *N *sequences, they are hence accessed in *O*(*N*). The AlignedSequenceContainer is derived from the VectorSequenceContainer class, and adds site access. Since data are stored as sequences, the access time for a site is in *O*(*N*) and the complete set of *L *sites is acessed in *O*(*N *× *L*). The VectorSiteContainer proposes an alternative implementation by storing sequences as sites instead of sequence objects. The sequence access is hence achieved in *O*(*L*), and the site access in *O*(1). All methods working on containers only deal with the SequenceContainer or the OrderedSequenceContainer interface, whith no assumption about the implementation. The execution time, however, may vary depending on the implementation used.

Development is facilitated by the use of the code documentation generated by the doxygen program [5]. Full class documentation can be consulted online or downloaded in navigable HTML format. A short tutorial is also available.

### Library content

We split Bio++ classes into five libraries: three main biological libraries (SeqLib, PhylLib and PopGen-Lib) and two utility libraries (Utils and NumCalc), see table 1 for there content and dependencies:

• the Utils library contains core classes and utilitary functions;

• the NumCalc library contains classes for numerical calculus, including several optimization tools, random number generators, probability distributions and statistical functions;

• the SeqLib library is dedicated to sequence analysis: sequence storage and manipulation, input/output toward several file formats, alphabet translation, etc.

• the PhylLib library deals with phylogenetic trees: Markov models, likelihood calculation, etc. The library is more focused on molecular evolution (model fitting, ancestral state reconstruction, across-site rate variation) than pure phylogenetic reconstruction, although several topology reconstruction methods are implemented (distance methods, nearest-neighbor interchanges (NNI) search for maximum likelihood (ML) and parsimony scores);

• the PopGenLib library is dedicated to population genetics, with particular sequence and codominant markers storage. Methods are provided for polymorphism analysis, population divergence estimation and neutrality tests.

## Results

**Figure 2:**
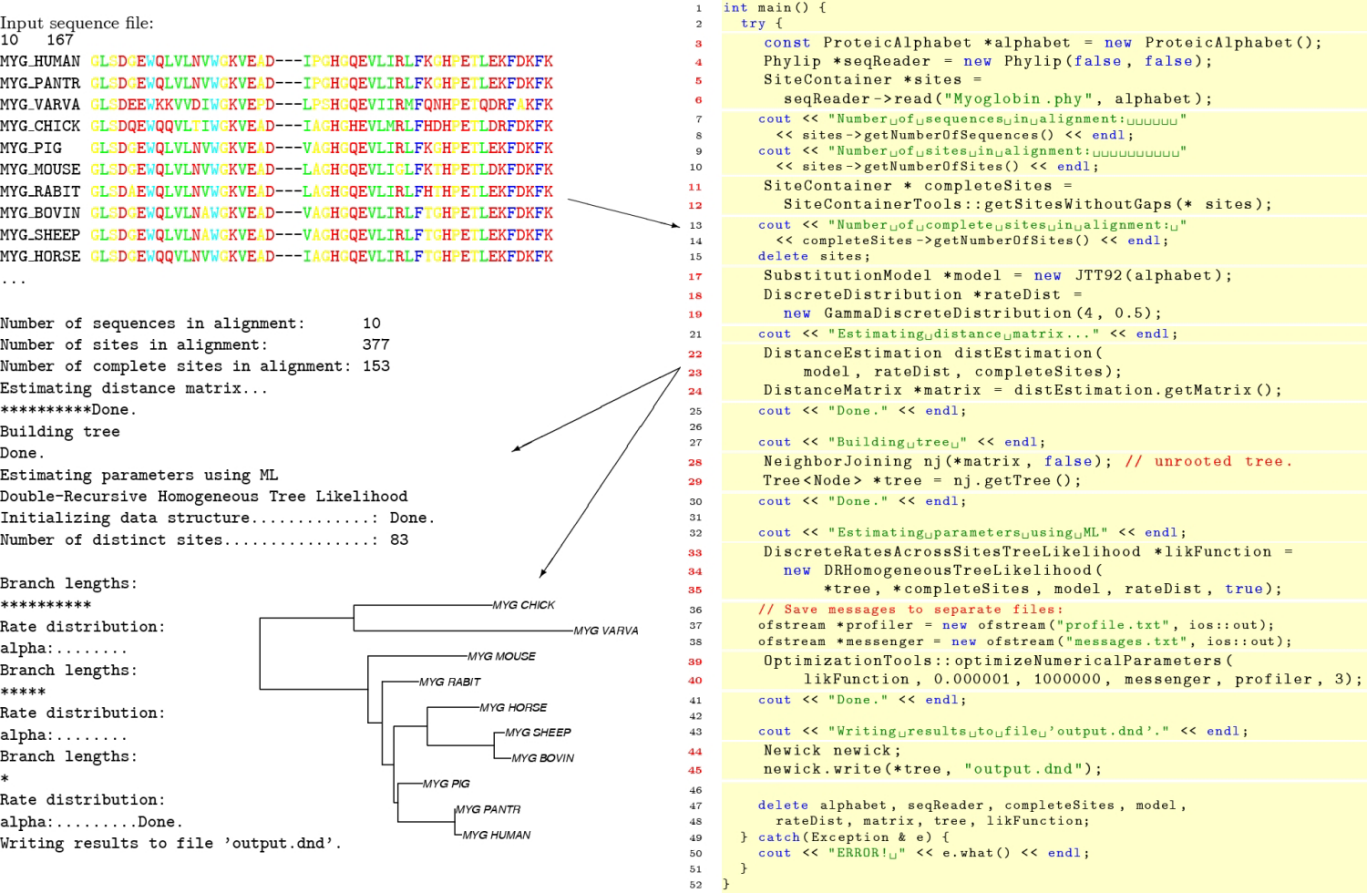
**Code example. **Lines numbered in red are referenced in the text.

Figure 2 shows a full program example. This application builds a Neighbor-Joining tree from a sequence file in Phylip format, and re-estimates parameters (branch lengths and shape of the rate across-site distribution) using maximum likelihood. The final tree is then written to a file in Newick format. Additional output files are also created, providing detailed information about the estimation procedure. The program begins with the creation of a ProteicAlphabet object (line 3):

const ProteicAlphabet * alphabet = new ProteicAlphabet();

This object specifies the type of sequence the program will use. The Alphabet object family contains classes for nucleic alphabets (DNA and RNA), proteins or codons (nuclear and mitochondrial, for vertebrates, echinoderms/nematodes and other invertebrates). A dataset is then readed from a Phylip sequence file. A sequence reader is created using

Phylip * seqReader = new Phylip(false, false);

Other file formats for reading and writing are supported, including the commonly used Fasta and Clustal. Sequences are then read and stored in a container:

SiteContainer * sites = seqReader->read("Myoglobin.phy", alphabet);

Since sequences are aligned, they are actually stored in a SiteContainer object (see figure 1). Line 7 to 10 display on screen some properties of the container, namely the number of sequences and sites. In this example we need to restrict our analysis to sites without any gap, so at line 11 a new container called "completeSites" is created from the former:

SiteContainer * completeSites = SiteContainerTools: :getSitesWithoutGaps(* sites);

Several {*}Tools classes providing utilitary functions are available. For instance, the SiteContainerTools class contains static functions that work on SiteContainer objects.

A JTT proteic SubstitutionModel object is created [6,7]:

SubstitutionModel * model = new JTT92(alphabet);

Here again, several commonly used models are available. We also use a discrete gamma rate across sites distribution with 4 rate classes and a shape parameter of 0.5 [8]:

DiscreteDistribution * rateDist = new GammaDiscreteDistribution(4, 0.5);

This model is used first to estimate the distance matrix from the data. A DistanceEstimation object is then created, by giving the model and data as parameters:

DistanceEstimation distEstimation(model, rateDist, completeSites);

DistanceMatrix * matrix = distEstimation.getMatrix();

Distances will be estimated using a maximum likelihood (ML) procedure. The NumCalc library provides an object-oriented implementation of several general optimization procedures (Optimizer objects). For phylogenetic optimization, specific optimizers are also available. The program then computes a neighbor-joining tree [9]. A Neighbor Joining object is instantiated and the resulting tree stored in a variable:

NeighborJoining nj(* matrix, false);

Tree<Node> * tree = nj.getTree();

This variable is passed to a TreeLikelihood object, together with the data set, model and rate distribution. An additional parameter tells the object to display a few informations. The DRHomogeneousTreeLikelihood object is for homogeneous models, with rate across sites variations.

DiscreteRatesAcrossSitesTreeLikelihood * likFunction = new DRHomogeneousTreeLikelihood(*tree, *completeSites, model, rateDist, true);

Several TreeLikelihood implementations are available. The DRHomogeneousTreeLikelihood object uses one 'view' by neighbor node for each node, *i.e. *three views by node in case of bifurcating trees [10]. This implementation saves computational time during NNI-mediated topology search, and is also more convenient to compute branch-lengths derivatives. An alternative implementation is proposed: HomogeneousTreeLikelihood, and others could be easily added if required. For parameter optimization, we used a utilitary function from the OptimizationTools class. This function automatically instanciates the appropriate optimizer object. It receives as argument a pointer toward the likelihood function, a tolerance number and a maximum number of function evaluations. Two optional log-files are provided to monitor the optimization process ("profiler" and "messenger", which contain several detailed informations, like for instance all parameters and function values at each step of the process), and the last parameter specifies the verbose level:

OptimizationTools:: optimizeNumericalParameters(likFunction, 0.000001, 1000000, messenger, profiler, 3);

Finally, the optimized tree is writen to a file named output. dnd in Newick file format:

Newick newick;

newick.write(*tree, "output.dnd");

This program creates three files: two for the ML tree estimation and one for the final tree.

## Conclusion

Bio++ is a mature project which has been used in previous works like molecular coevolution analysis [11] or codon analysis [12]. However it is an active project still receiving new methods and improvements.

Development snapshots may be accessed by anonymous CVS on the library website. The website also provides on-line documentation of classes, tutorial and several code examples. Several programs have been developped with the Bio++ libraries, including bppML for ML likelihood tree estimation and bppSG for sequence generation by simulation under different kinds of models. Any contribution will be welcome, as specific functions or as additional libraries compatible with the present ones.

## Availability and requirements

C++ libraries are not organized in a tree-like hierarchy as java packages or perl modules. They are bundled in a non-nested way, and may be compiled in two flavours, either static or shared (= dynamic). Dynamic libraries are loaded during the program execution and hence can be shared by several applications, while static libraries are hard-coded into the executable, which no longer requires the library to be installed.

Each Bio++ library is complient with the GNU standards and uses the autotools suite for compilation and installation. Bio++ can hence be built with these GNU tools on any unix-like system. Alternatively, sources can be imported and compiled in any C++ development environment (IDE). It has been successfully installed on Linux, MacOS X and Windows using the Cygwin port and MinGW. Bio++ is distributed freely under the CeCILL public license (the French free software license, compatible with the GNU General Public License). It is available from its website at .

## Authors' contributions

JD developped the Utils, NumCalc, SeqLib and PhylLib libraries and drafted the manuscript. SGa developped the PopGenLib library, provided algorithms for the NumCalc library and helped with the GNU configuration tools. EB developped tools for the PopGenLib library. SG1 provided tools for the PopGenLib and SeqLib libraries. VR helped in the development of the PhylLib library. NG participated in the design of the library. KB suppervised the whole project. SG1, VR, NG and KB helped to draft the manuscript. All authors read and approved the final manuscript.

## Supplementary Material

Additional File 1 Sources and data files of the example as a zipped archive. Click here for file

## Figures and Tables

**Table 1:** Libraries available and dependencies.

Library	Utils	NumCalc	SeqLib	Content
Utils				Basal classes and utilities. Exceptions, text and file manipulation.
NumCalc				Numerical calculus. Vector and matrix manipulation, optimization, algebra, probability distribution and statistics, random number generation.
SeqLib				Sequence manipulation, alphabets, chemical properties and distances, input/output in various formats (Fasta, Phylip, Clustal, etc.).
PhylLib				Phylogenetics. Tree reconstruction by maximum parsimony, distance methods or maximum likelihood, parameter estimations, ancestral states reconstruction, etc.
PopGenLib				Population genetics. Polymorphism statistics, linkage desequilibrium, neutrality tests and recombination.
